# A New Dental College

**Published:** 1878-06

**Authors:** 


### A NEW DENTAL COLLEGE.

We have received the first annual announcement of the dental department of the University of Pennsylvania, 1878-9.

This institution organized in connection with and under the protecting care of an old, well established and popular university, will at once have resources and elements of strength, that a new institution without such connection, could not attain except through long time and much effort and labor on the part of projectors and conductors.

If this department is put upon an equal footing with the other departments of the University, it will be successful; but if it is to be a mere parasite, it would be better it had never been organized.

The faculty is composed of teachers of large experience and designedly high reputation, and in their respective work there will be no failure.

There are advantages accruing to a dental college in such a connection not to be lightly esteemed.

It brings to its aid and support the large and extended resources of the university. It renders practicable great economy in the working force, in appliances, apparatus, buildings, etc., for teaching.

If a good knowledge of the basal principles of medicine and surgery is important as a foundation for a thorough dental education, the university can more easily and thoroughly supply this requirement.

The salary of the teacher in the university college is not contingent upon the number of students in attendance. It would be preferable in all educational departments, that the teacher be placed outside the influence of student fees.

It is within the power and practicable for the university to establish rules, requirements and a curriculum that can hardly be done elsewhere. The university plan of education will not become universal and supersede all others, yet there is a growing desire to test its capabilities; at least in many departments of education.

